# Associations of physical activity type, intensity, and frequency with subclinical hypothyroidism: a cross-sectional analysis of NHANES 2007–2012

**DOI:** 10.3389/fpubh.2025.1499070

**Published:** 2025-07-07

**Authors:** Zeyu Li, Yu Mao, Xiaoyong Wen, Guangji Chen, Shiwei Zhou

**Affiliations:** ^1^Department of Thyroid Surgery, The Affiliated Cancer Hospital of Xiangya School of Medicine, Central South University/Hunan Cancer Hospital, Changsha, Hunan, China; ^2^Department of Thyroid Surgery, The Second Xiangya Hospital, Central South University, Changsha, Hunan, China; ^3^Department of Surgery, University Hospital, Central South University, Changsha, Hunan, China

**Keywords:** subclinical hypothyroidism, physical activity, moderate to vigorous physical activity, vigorous physical activity, occupational physical activity, NHANES

## Abstract

**Objective:**

Subclinical hypothyroidism (SCH) is a prevalent endocrine disorder associated with metabolic and cardiovascular risks. While physical activity (PA) is well recognized for its benefits on metabolic and cardiovascular health, its relationship with SCH remains unclear. This study aimed to examine the associations between different types, intensities, and frequencies of PA and SCH prevalence using nationally representative data from the U. S. National Health and Nutrition Examination Survey (NHANES).

**Methods:**

Data from the 2007–2012 NHANES cycles were analyzed. Weighted univariate and multivariate logistic regression models were employed to assess the associations between PA and SCH prevalence. Curve fitting and threshold effect analyses were conducted to explore potential non-linear relationships, while subgroup analyses examined effect modifications by demographic and clinical factors.

**Results:**

Among 6,133 participants included in the final analysis (approximately 20.15% of the total NHANES sample), the prevalence of SCH was 2.5%. Individuals without SCH exhibited significantly higher total PA duration, particularly in occupational physical activity (OPA), vigorous physical activity (VPA), moderate-to-vigorous physical activity (MVPA), MVPA intensity, and weekly frequency of vigorous occupational physical activity (VOPA). In the fully adjusted model, each 10-h/week increase in VPA and MVPA was associated with 33% (OR = 0.67, 95% CI: 0.49–0.91) and 11% (OR = 0.89, 95% CI: 0.81–0.98) lower odds of SCH, respectively. A 10% increase in MVPA intensity was linked to an 8% reduction (OR = 0.92, 95% CI: 0.87–0.97). Similar inverse associations were found for PA and OPA (PA: OR = 0.90, 95% CI: 0.82–0.98; OPA: OR = 0.90, 95% CI: 0.81–0.99). Among PA frequency measures, only VOPA frequency was significant, with each additional session per week associated with a 17% reduction in odds (OR = 0.83, 95% CI: 0.74–0.94). Curve fitting analysis revealed a non-linear relationship between MVPA intensity and SCH prevalence, with a threshold at 57.14%. Below this threshold, higher MVPA intensity was associated with lower SCH prevalence (*p* = 0.001), whereas above this threshold, the association became non-significant. Subgroup analyses identified a significant interaction with age, where the protective effects of PA and OPA were significant only in individuals aged <60 years.

**Conclusion:**

This study suggests that higher PA levels, particularly at greater intensities and frequencies, are associated with a lower prevalence of SCH, especially in individuals aged <60 years. These findings highlight the potential role of regular, high-intensity PA in reducing SCH risk.

## Introduction

Subclinical hypothyroidism (SCH), characterized by elevated thyroid-stimulating hormone (TSH) levels alongside normal free thyroxine (FT4) levels, is a prevalent endocrine disorder affecting up to 10% of adults, with the highest prevalence observed in women and the older adult (1, 2). SCH has been associated with various metabolic and cardiovascular complications, including elevated serum total cholesterol, low-density lipoprotein cholesterol (LDL-C), and triglycerides (TG), as well as an increased risk of cardiovascular disease and cognitive impairment (3). Given its potential long-term health consequences, understanding modifiable risk factors that may influence SCH prevalence is of significant clinical and public health interest.

Physical activity (PA), defined as any bodily movement that results in energy expenditure, is typically categorized into occupational physical activity (OPA), transportation physical activity (TPA), and leisure-time physical activity (LTPA), with further classification based on intensity into moderate physical activity (MPA) and vigorous physical activity (VPA) (4, 5). While PA is well known for its beneficial effects on metabolic and cardiovascular health, its relationship with thyroid function remains less clear. A population-based cohort study reported no significant association between total PA and thyroid function (as defined by TSH and FT4 levels) (6), whereas other studies have suggested that PA may influence thyroid-related biochemical markers and thyroid diseases (7).

For instance, previous research has demonstrated that PA significantly impacts thyroid function, particularly among military personnel and athletes, where intense physical activity may alter hormonal homeostasis (8). Wu et al. (9) carried out a cross-sectional, community-based study in Fujian Province, China, including 318 individuals (159 with SCH and 159 euthyroid controls), and observed that PA was closely associated with thyroid hormone levels and homeostasis. However, both studies were based on small, non-representative samples, limiting their generalizability to broader populations. These limitations highlight the need for large, population-based studies such as those using NHANES data to better understand the PA–thyroid function relationship in the general U. S. population.

Despite growing interest in the potential role of PA in thyroid health, there is a lack of large-scale studies systematically examining the associations between different PA types, intensities, and frequencies and SCH prevalence in the general population. Given PA’s potential involvement in metabolic regulation, inflammation reduction, and endocrine homeostasis, we hypothesize that higher PA levels, particularly VPA and frequent PA engagement, are inversely associated with SCH prevalence. Furthermore, we predict that this association may vary by PA type (OPA, TPA, LTPA) and intensity (MPA vs. VPA), with vigorous and occupational PA exhibiting stronger protective effects.

To test this hypothesis, we utilized data from the National Health and Nutrition Examination Survey (NHANES) database, adjusting for potential confounders, to comprehensively explore the associations between PA patterns and SCH prevalence in a nationally representative population.

## Methods

### NHANES description and ethical approval

The NHANES is a comprehensive, nationally representative survey conducted in the United States that collects data on demographics, health-related behaviors, and nutritional status. All survey protocols were reviewed and approved by the Ethics Review Board of the National Center for Health Statistics (NCHS), in accordance with the ethical standards of institutional and national research committees and the 1964 Declaration of Helsinki and its later amendments. All participants provided written informed consent prior to participation. NHANES data are publicly available online.^1^

### Study participants

**Figure 1:**
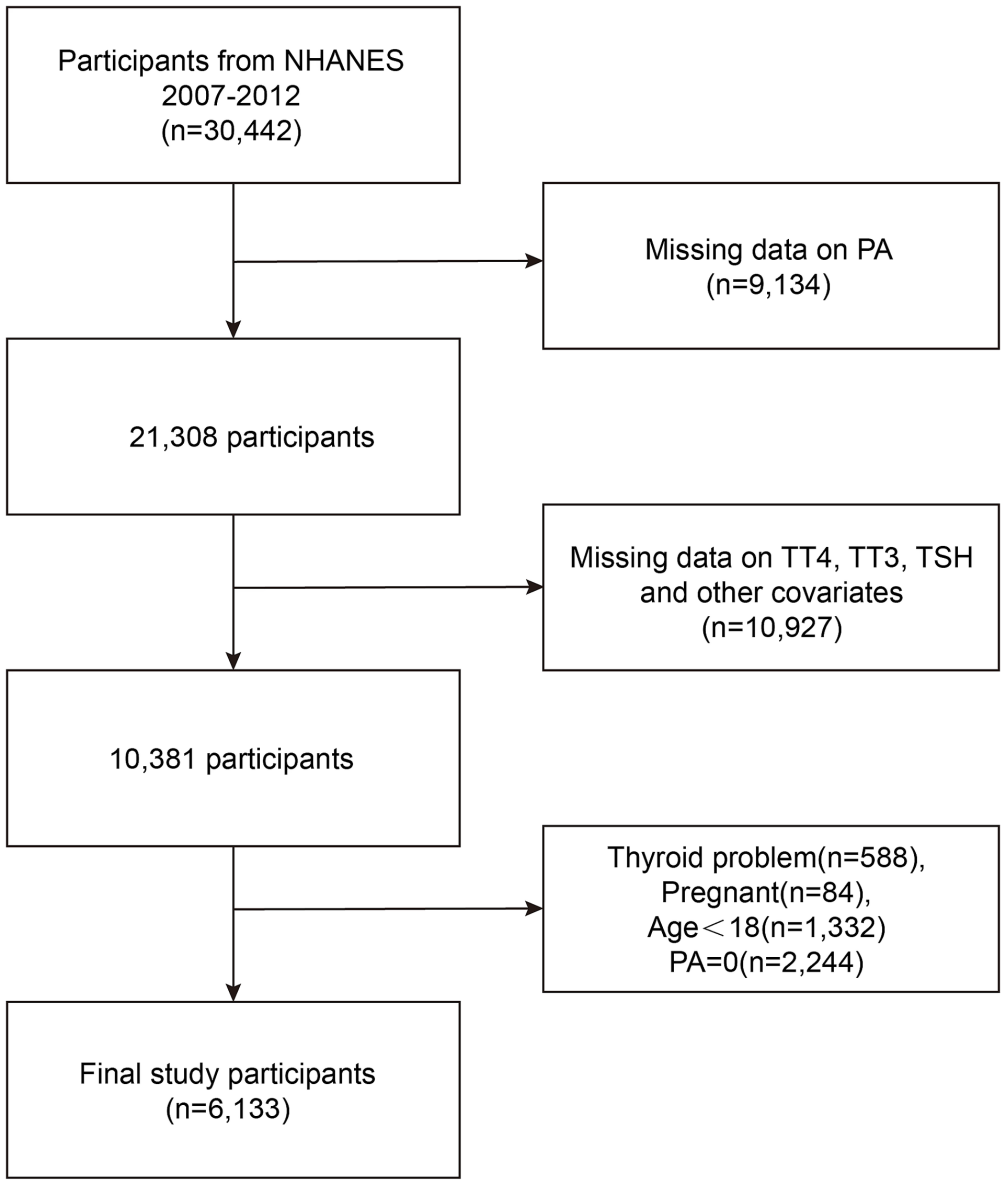
Flowchart of participant selection.

Participants were drawn from the NHANES 2007–2008, 2009–2010, and 2011–2012 cycles, comprising a nationally representative sample of 30,442 individuals. For this study, we included adults aged 18 years or older with available thyroid function measurements and complete data on PA assessments. We excluded individuals with missing PA data (*N* = 9,134), those lacking laboratory data necessary for the diagnosis of subclinical hypothyroidism (SCH) (*N* = 10,927), individuals with known thyroid disorders (*N* = 588) to minimize potential confounding, pregnant participants (*N* = 84) due to pregnancy-related hormonal changes, and participants reporting zero PA (*N* = 2,244) to avoid extreme outliers and better assess dose–response relationships. After applying these criteria, the final analytical sample consisted of 6,133 participants (Figure 1).

### Definition and measurement of SCH

The SCH was defined as a TSH level ≥ 4.5 mIU/L with an FT4 level within the normal range of 9–25 pmol/L (10). Serum total thyroxine, total triiodothyronine, and free triiodothyronine levels were measured using a competitive binding immunoenzymatic assay. Serum FT4 was measured using a two-step immunoenzymatic assay, and serum TSH was measured using a third-generation two-site immunoenzymatic (sandwich) assay. Thyroid function tests were conducted only among participants aged ≥12 years who met NHANES laboratory eligibility criteria.

### Definition of PA

PA data were collected from all participants aged ≥2 years. PA was self-reported by participants using the Global Physical Activity Questionnaire (4). PA was categorized into three common domains: OPA, TPA, and LTPA. Participants reported the frequency, intensity, and duration of PA in a typical week, distinguishing between vigorous and moderate PA for both OPA and LTPA. For OPA or LTPA, minutes spent in VPA were doubled and added to the minutes of MPA. This total was then multiplied by the number of active days to determine the total PA minutes per week (4). To account for variability in PA values, PA was converted from minutes to 10-h units. The intensity of moderate-to-vigorous physical activity (MVPA) was calculated as the proportion of VPA to total MVPA. For example, a person engaging in 40 min of VPA and 80 min of MPA in a typical week would accumulate 160 min of MVPA, with VPA accounting for 50% of the total MVPA (11).

### Covariates

The analysis adjusted for a comprehensive set of covariates encompassing demographic, lifestyle, and clinical factors. Demographic variables included age, gender (male or female), race/ethnicity (non-Hispanic White, non-Hispanic Black, Mexican American, and other races), educational attainment (less than high school, high school graduate or GED, and more than high school), family poverty level (categorized by family poverty income ratio as <1.3, 1.3–3.5, and ≥3.5), and marital status (married or cohabitating, widowed/divorced/separated, and never married). Anthropometric and clinical variables included body mass index (BMI), classified as <25, 25–30, and ≥30 kg/m^2^, as well as hypertension and diabetes, both defined by either a physician diagnosis or the use of related medications. Lifestyle-related covariates comprised smoking status (defined as having smoked ≥100 cigarettes in a lifetime), heavy alcohol consumption (≥5 drinks per day), average daily sedentary time (hours), and average sleep duration (hours). Urinary iodine concentration (UIC), a biological indicator relevant to thyroid function, was also included and categorized as <100 μg/L, 100–300 μg/L, and ≥300 μg/L, measured via inductively coupled plasma dynamic reaction cell mass spectrometry. Sedentary behavior was further assessed based on self-reported sitting time during activities such as reading, watching television, computer use, or traveling in a vehicle.

### Statistical analysis

Statistical analyses were performed using R software (R Foundation: http://www.r-project.org; version 3.4.3) and Empower (R) (www.empowerstats.com, X&Y Solutions, Inc., Boston, Massachusetts). Given the complex, multistage sampling design of NHANES, MEC examination weights were applied to ensure representativeness and account for survey design effects.

Categorical variables were expressed as absolute frequencies (n) with weighted percentages, while continuous variables were presented as weighted means ± standard deviations. Differences between groups were assessed using Chi-square tests for categorical variables, while parametric tests (*t*-tests, ANOVA) were employed for normally distributed continuous variables, and non-parametric tests (Mann–Whitney *U*, Kruskal–Wallis) were applied for non-normally distributed data. All statistical tests were two-sided, with a significance threshold of *p* < 0.05.

To examine the association between PA and SCH prevalence, multivariable logistic regression models were constructed. Model 1 was unadjusted, while Model 2 accounted for gender, age, and race. Model 3 further adjusted for education level, marital status, FPL, BMI, alcohol consumption, smoking status, hypertension, diabetes, UIC, sedentary time, and sleep duration.

Potential non-linear associations were explored using smoothing curve fitting, where the central line represented the effect size and the shaded areas denoted the 95% confidence interval (CI). In addition, threshold effect analysis was conducted to identify possible cutoff points in the association between MVPA intensity and SCH prevalence.

Subgroup analyses were performed to assess the associations across various demographic and clinical strata, while interaction effects were evaluated using multiplicative interaction terms. The statistical significance of these effect modifications was determined using *p*-values for interaction.

## Results

This study included 6,133 participants from the NHANES 2007–2012 datasets, with an average age of 43.18 ± 16.15 years. Among them, 54.57% were male and 45.43% were female.

### PA differences

Significant differences in PA parameters were observed between participants with SCH and euthyroid individuals. Participants in the euthyroid group exhibited higher levels of VPA and MVPA compared to those with SCH. Specifically, individuals in the euthyroid group had significantly greater mean values for VPA per 10 h (0.55 ± 1.05 vs. 0.30 ± 0.76, *p* = 0.001), MVPA per 10 h (1.99 ± 2.73 vs. 1.45 ± 2.01, *p* = 0.009), MVPA intensity per 10% (3.60 ± 3.86 vs. 2.42 ± 3.57, *p* < 0.0001), total PA per 10 h (2.19 ± 2.85 vs. 1.66 ± 2.12, *p* = 0.014), OPA per 10 h (1.51 ± 2.63 vs. 1.00 ± 1.84, *p* = 0.009), and weekly frequency of VOPA (1.16 ± 2.07 vs. 0.65 ± 1.70, *p* = 0.001). However, no significant differences were found between the two groups in MPA, TPA, LTPA, or in the frequencies of MVPA, moderate LTPA, vigorous LTPA, and TPA. Additionally, sedentary time, sleep duration, and UIC did not significantly differ between groups (Table 1).

**Table 1:** Baseline characteristics stratified by SCH.

Characteristic^a^	Euthyroid	SCH	*P*-value
Age (years)	43.13 ± 16.12	45.05 ± 17.07	0.115
Gender, *n* (%)			0.844
Male	3,331 (54.60)	88 (53.86)	
Female	2,647 (45.41)	67 (46.15)	
Race, *n* (%)			<0.001
Non-Hispanic White	2,718 (69.98)	102 (84.29)	
Non-Hispanic Black	1,194 (10.23)	10 (2.75)	
Mexican American	975 (8.13)	17 (4.69)	
Other Race	1,091 (11.66)	26 (8.27)	
Education, *n* (%)			0.409
Less than high school diploma	1,570 (17.47)	47 (17.99)	
High school graduate/GED	1,392 (23.24)	35 (27.19)	
More than high school diploma	3,016 (59.29)	73 (54.82)	
Marital status, *n* (%)			0.042
Married or cohabitating	3,410 (64.45)	95 (72.21)	
Widowed, divorced, or separated	1,116 (19.99)	19 (12.42)	
Never married	1,085 (15.56)	34 (15.37)	
FPL, *n* (%)			0.447
<1.3	1,695 (20.44)	48 (21.04)	
1.3–3.5	2003 (33.48)	52 (37.53)	
≥3.5	1776 (46.09)	41 (41.42)	
BMI (kg/m^2^), *n* (%)			0.591
<25	1972 (35.4)	43 (35.73)	
25–30	2034 (34.61)	53 (31.33)	
≥30	1912 (30)	59 (32.94)	
Alcohol consumption, *n* (%)			0.775
No	1,237 (19)	33 (18.12)	
Yes	4,003 (80)	109 (81.88)	
Hypertension, *n* (%)			0.248
No	4,302 (74.72)	33 (70.92)	
Yes	1,676 (25.28)	109 (29.08)	
Diabetes, *n* (%)			0.840
No	5,427 (93.54)	139 (93.92)	
Yes	551 (6.46)	16 (6.08)	
Smoking, *n* (%)			0.004
No	2,993 (53.46)	93 (64.71)	
Yes	2,615 (46.54)	55 (35.29)	
UIC (μg/L), *n* (%)			0.316
<100	1926 (33.28)	44 (30.77)	
100–300	2,835 (48.5)	73 (46.58)	
≥300	1,069 (18.22)	33 (22.65)	
Sleep duration (h)	6.82 ± 1.30	6.89 ± 1.36	0.473
Sedentary time (h)	5.49 ± 3.23	5.61 ± 3.35	0.638
PA (10 h/week)	2.19 ± 2.85	1.66 ± 2.12	0.014
OPA (10 h/week)	1.51 ± 2.63	1.00 ± 1.84	0.009
TPA (10 h/week)	0.20 ± 0.59	0.21 ± 0.57	0.785
LTPA (10 h/week)	0.48 ± 0.75	0.45 ± 0.67	0.632
MPA (10 h/week)	0.89 ± 1.25	0.86 ± 1.10	0.745
VPA (10 h/week)	0.55 ± 1.05	0.30 ± 0.76	0.001
MVPA (10 h/week)	1.99 ± 2.73	1.45 ± 2.01	0.009
MVPA intensity (10%/week)	3.60 ± 3.86	2.42 ± 3.57	<0.0001
Moderate OPA frequency (times/week)	2.29 ± 2.52	2.44 ± 2.51	0.412
Vigorous OPA frequency (times/week)	1.16 ± 2.07	0.65 ± 1.70	0.001
TPA frequency (times/week)	1.63 ± 2.57	1.62 ± 2.54	0.936
Moderate LTPA frequency (times/week)	1.84 ± 2.12	2.11 ± 2.21	0.096
Vigorous LTPA frequency (times/week)	1.10 ± 1.81	1.01 ± 1.97	0.510
MVPA frequency (times/week)	6.38 ± 4.41	6.21 ± 4.52	0.601

^a^Data are presented as *n* (%) or mean ± standard deviation.

SCH, subclinical hypothyroidism; FPL, family income to poverty ratio; BMI, body mass index; UIC, urinary iodine concentration; PA, physical activity; OPA, occupational physical activity; TPA, transportation-related physical activity; LTPA, leisure-time physical activity; MPA, moderate physical activity; VPA, vigorous physical activity; MVPA, moderate-to-vigorous physical activity.

### Association between PA and SCH

Logistic regression analyses revealed a significant inverse association between PA and SCH prevalence. In the unadjusted model, the odds ratio (OR) for SCH per 10-h increase in PA time was 0.92 (95% CI: 0.86–0.99, *p* = 0.023), and per 10-h increase in OPA time, the OR was 0.92 (95% CI: 0.85–0.99, *p* = 0.033). These findings suggest that higher PA levels, particularly in occupational settings, are associated with lower SCH prevalence (Table 2).

**Table 2:** Relationship between various PA and SCH, showed by weighted multivariate logistic regression.

SCH	Model 1	*P*-value	Model 2	*P*-value	Model 3	*P*-value
	OR (95% CI)		OR (95% CI)		OR (95% CI)	
PA (10 h/week)	0.92 (0.86, 0.99)	0.023	0.93 (0.86, 1.00)	0.048	0.90 (0.82, 0.98)	0.022
OPA (10 h/week)	0.92 (0.85, 0.99)	0.033	0.92 (0.85, 1.00)	0.043	0.90 (0.81, 0.99)	0.034
TPA (10 h/week)	0.94 (0.71, 1.25)	0.680	1.00 (0.77, 1.31)	0.998	0.97 (0.71, 1.31)	0.829
LTPA (10 h/week)	0.90 (0.71, 1.14)	0.374	0.96 (0.76, 1.21)	0.714	0.82 (0.58, 1.17)	0.274

Model 1 was adjusted for none. Model 2 was adjusted for gender, age, and race. Model 3 was adjusted for gender, age, race, education, marital status, FPL, BMI, alcohol consumption, smoking, hypertension, diabetes, UIC, sedentary time, and sleep duration.

PA, physical activity; SCH, subclinical hypothyroidism; OPA, occupational physical activity; TPA, transportation-related physical activity; LTPA, leisure-time physical activity; FPL, family income to poverty ratio; BMI, body mass index; UIC, urinary iodine concentration; CI, confidence interval; OR, odds ratio.

Further analysis of PA intensity demonstrated that increased engagement in VPA and MVPA was linked to a reduced likelihood of SCH. Specifically, per 10-h increase in VPA, the OR for SCH was 0.74 (95% CI: 0.59–0.93, *p* = 0.01), while for MVPA time, the OR was 0.92 (95% CI: 0.85–0.99, *p* = 0.023). Additionally, each 10% increase in MVPA intensity was associated with a significant reduction in SCH prevalence (OR = 0.92, 95% CI: 0.88–0.96, *p* < 0.001). These results indicate that engaging in high-intensity PA may be particularly beneficial in reducing SCH risk (Table 3).

**Table 3:** Relationship between moderate to vigorous PA and SCH, showed by weighted multivariate logistic regression.

SCH	Model 1	*P*-value	Model 2	*p*-value	Model 3	*P*-value
	OR (95% CI)		OR (95% CI)		OR (95% CI)	
MPA (10 h/week)	0.96 (0.84, 1.10)	0.540	0.96 (0.83, 1.10)	0.520	0.94 (0.80, 1.11)	0.490
VPA (10 h/week)	0.74 (0.59, 0.93)	0.010	0.76 (0.60, 0.96)	0.022	0.67 (0.49, 0.91)	0.011
MVPA (10 h/week)	0.92 (0.85, 0.99)	0.023	0.92 (0.85, 1.00)	0.040	0.89 (0.81, 0.98)	0.020
MVPA intensity (10%/week)	0.92 (0.88, 0.96)	<0.001	0.93 (0.88, 0.97)	0.003	0.92 (0.87, 0.97)	0.004

Model 1 was adjusted for none. Model 2 was adjusted for gender, age, and race. Model 3 was adjusted for gender, age, race, education, marital status, FPL, BMI, alcohol consumption, smoking, hypertension, diabetes, UIC, sedentary time, and sleep duration.

PA, physical activity; SCH, subclinical hypothyroidism; MPA, moderate physical activity; VPA, vigorous physical activity; MVPA, moderate-to-vigorous physical activity; FPL, family income to poverty ratio; BMI, body mass index; UIC, urinary iodine concentration; CI, confidence interval; OR, odds ratio.

Regarding PA frequency, only VOPA frequency was significantly associated with lower odds of SCH (OR = 0.87, 95% CI: 0.79–0.96, *p* = 0.006) (Table 4).

**Table 4:** Relationship between the frequency of various PA and SCH, showed by weighted multivariate logistic regression.

SCH	Model 1	*P*-value	Model 2	*P*-value	Model 3	*P*-value
	OR (95% CI)		OR (95% CI)		OR (95% CI)	
Moderate OPA frequency (times/week)	1.02 (0.96, 1.09)	0.509	1.01 (0.95, 1.08)	0.727	1.01 (0.94, 1.09)	0.769
Vigorous OPA frequency (times/week)	0.87 (0.79, 0.96)	0.006	0.87 (0.78, 0.96)	0.008	0.83 (0.74, 0.94)	0.004
TPA frequency (times/week)	0.98 (0.93, 1.05)	0.700	1.01 (0.95, 1.07)	0.796	1.02 (0.95, 1.09)	0.646
Moderate LTPA frequency (times/week)	1.06 (0.99, 1.14)	0.076	1.04 (0.97, 1.12)	0.244	1.01 (0.93, 1.09)	0.870
Vigorous LTPA frequency (times/week)	0.94 (0.85, 1.04)	0.224	0.98 (0.88, 1.08)	0.634	1.00 (0.89, 1.13)	0.974
MVPA frequency (times/week)	0.99 (0.96, 1.03)	0.614	0.99 (0.95, 1.03)	0.533	0.98 (0.93, 1.02)	0.286

Model 1 was adjusted for none. Model 2 was adjusted for gender, age, and race. Model 3 was adjusted for gender, age, race, education, marital status, FPL, BMI, alcohol consumption, smoking, hypertension, diabetes, UIC, sedentary time, and sleep duration.

PA, physical activity; SCH, subclinical hypothyroidism; OPA, occupational physical activity; TPA, transportation-related physical activity; LTPA, leisure-time physical activity; MVPA, moderate-to-vigorous physical activity; FPL, family income to poverty ratio; BMI, body mass index; UIC, urinary iodine concentration; CI, confidence interval; OR, odds ratio.

### Adjustment for confounders

To ensure robustness, further adjustments were made in Model 2 and Model 3. The significant negative associations between PA and SCH prevalence remained consistent across all models (Tables 2–4).

### Non-linear relationship between MVPA intensity and SCH

**Figure 2:**
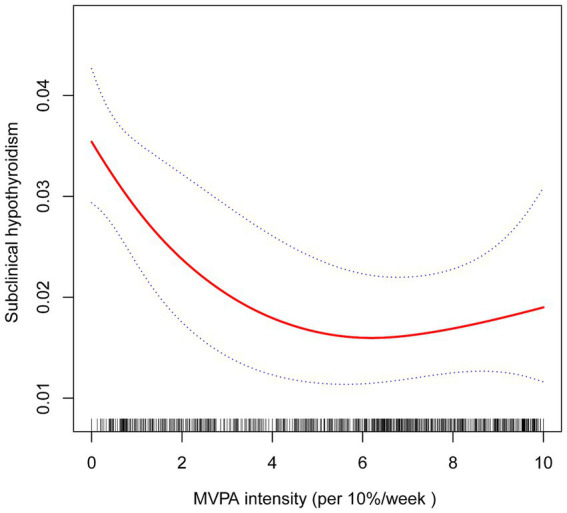
The association between MVPA intensity and SCH. The solid red line represents the estimated value and the blue dashed areas indicate their corresponding 95% confidence interval. Adjustment factors included gender, age, race, education, marital status, FPL, BMI, alcohol consumption, smoking, hypertension, diabetes, UIC, sedentary time, and sleep duration.

A non-linear association between MVPA intensity and SCH prevalence was identified using smooth curve fitting and threshold effect analysis. A generalized additive model revealed a critical threshold for MVPA intensity at 57.14% (*K* = 5.714). Below this threshold (*K* < 5.714), a significant negative association was observed between MVPA intensity and SCH prevalence (OR = 0.82, 95% CI: 0.72–0.93, *p* = 0.002, per 10% increase in intensity). Conversely, above this threshold (*K* ≥ 5.714), the association was positive but non-significant (OR = 1.16, 95% CI: 0.93–1.45, *p* = 0.180, per 10% increase in intensity), with a log-likelihood ratio test value of 0.03 (Figure 2 and Table 5).

**Table 5:** Analysis of MVPA intensity and SCH using segmented linear regression.

Models	OR (95% CI)	*P*-value
Model 1		
One line effect	0.92 (0.87, 0.97)	0.004
Model 2		
Inflection point (K)	5.714	
<K	0.82 (0.72, 0.93)	0.002
>K	1.16 (0.93, 1.45)	0.180
*P* for log-likelihood ratio test		0.030

Model 1, one-line linear regression; Model 2, two-segment regression.

Adjust for: gender, age, race, education, marital status, FPL, BMI, alcohol consumption, smoking, hypertension, diabetes, UIC, sedentary time, and sleep duration.

MVPA, moderate-to-vigorous physical activity; SCH, subclinical hypothyroidism; FPL, family income to poverty ratio; BMI, body mass index; UIC, urinary iodine concentration; CI, confidence interval; OR, odds ratio.

### Subgroup analyses

**Figure 3:**
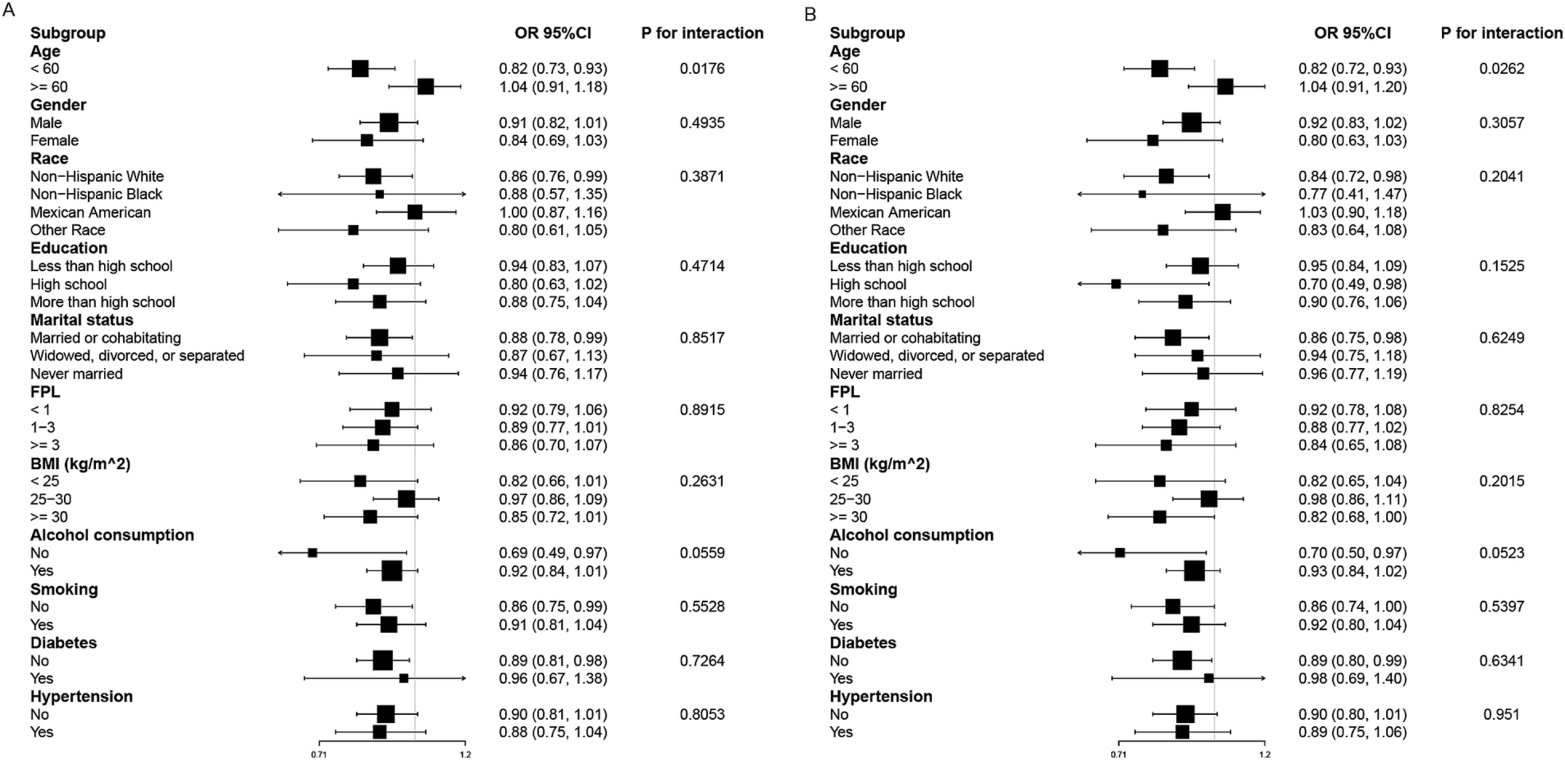
Subgroup analysis of PA **(A)**, OPA **(B)** and SCH. Adjustment factors included gender, age, race, education, marital status, FPL, BMI, alcohol consumption, smoking, hypertension, diabetes, UIC, sedentary time, and sleep duration.

Subgroup analyses were conducted to examine the association between PA, OPA, and SCH across different demographic and clinical strata (Figure 3). A significant interaction with age was observed in both PA (*P*-interaction = 0.0176) and OPA (*P*-interaction = 0.0262) analyses. The protective effect of higher PA and OPA was significant only in individuals aged <60 years (PA: OR = 0.82, 95% CI: 0.73–0.93; OPA: OR = 0.82, 95% CI: 0.72–0.93), whereas in those aged ≥60 years, no significant association was detected (PA: OR = 1.04, 95% CI: 0.91–1.18; OPA: OR = 1.04, 95% CI: 0.91–1.20). No significant interactions were found for gender, race, education, marital status, FPL, BMI, alcohol consumption, smoking, hypertension, or diabetes in either analysis (all *P*-interaction > 0.05).

## Discussion

The findings of this study, based on data from the NHANES 2007–2012 cycles, underscore the complex relationship between PA and SCH prevalence. Our analysis revealed that euthyroid participants exhibited significantly higher levels of total PA, OPA, VPA, MVPA, MVPA intensity, and weekly frequency of VOPA compared to those with SCH. These associations were further supported by multiple regression analyses, which demonstrated that higher VPA time, MVPA time, and MVPA intensity were significantly associated with lower odds of SCH prevalence. Importantly, these associations remained robust even after adjusting for potential confounders, suggesting that PA, particularly at higher intensities, may play a protective role against SCH development. Furthermore, subgroup analyses identified a significant interaction with age, where the protective effects of PA and OPA were significant only in individuals aged <60 years.

### The protective role of OPA

Among different PA types, OPA was significantly and inversely associated with SCH prevalence, suggesting that individuals engaged in physically demanding occupations, such as construction, mining, farming, and forestry, may have a lower risk of SCH. One possible explanation is that sustained occupational exertion enhances metabolic efficiency and reduces chronic low-grade inflammation, both of which are known to influence thyroid function. Additionally, frequent engagement in VOPA exhibited a stronger protective effect, implying that consistent and high-intensity work-related PA may be more beneficial than sporadic or low-intensity activities.

The protective role of OPA in metabolic health has been observed in other disorders as well. For instance, a cohort study in a Chinese population found that higher OPA levels were associated with a reduced risk of diabetes (12). Similarly, a meta-analysis by Aune et al. (13) reported that high-intensity OPA, compared to low-intensity OPA, was associated with a 15% lower risk of developing type 2 diabetes (RR = 0.85, 95% CI: 0.79–0.92). While the relationship between OPA and SCH remains underexplored, these findings suggest that OPA may confer broad metabolic benefits that could extend to thyroid health.

### Higher-intensity PA and its impact on SCH prevalence

Another key observation was that higher-intensity PA, particularly VPA, was associated with a greater reduction in SCH prevalence compared to MPA. Our findings demonstrated that each 10-h increase in VPA was associated with a 26% reduction in SCH prevalence, while MVPA time and MVPA intensity were associated with an 8.4 and 8.2% reduction, respectively. These findings reinforce the hypothesis that higher-intensity PA exerts stronger protective effects on thyroid function than lower-intensity PA. One plausible explanation is that vigorous exercise enhances mitochondrial efficiency (14), improves insulin sensitivity (15), and reduces systemic inflammation (16), all of which are implicated in thyroid dysfunction. This suggests that targeted interventions focusing on increasing PA intensity could be beneficial for individuals at risk of SCH.

### Non-linear relationship between MVPA intensity and SCH

This study also identified a non-linear relationship between MVPA intensity and SCH prevalence, as revealed by smooth curve fitting analysis. A critical threshold was observed at 57.14% MVPA intensity (*K* = 5.714). Below this threshold, higher MVPA intensity was significantly associated with lower SCH prevalence. However, beyond this point, the relationship became non-significant, suggesting the existence of an optimal range of MVPA intensity for reducing SCH risk.

The observed non-linear association implies that while moderate increases in MVPA intensity provide substantial protective benefits against SCH, exceeding a certain intensity level may yield diminishing returns or even pose potential risks. This pattern aligns with prior research indicating the presence of an optimal “sweet spot” in PA levels, where health benefits are maximized without introducing adverse effects (17–19). For instance, excessive VPA has been linked to increased risks of musculoskeletal injuries, cardiovascular strain, and overtraining syndrome, which could paradoxically impair overall health. Therefore, while promoting increased PA, it is essential to advocate for a balanced approach that considers individual capacities, health status, and the potential risks associated with excessive intensity.

### Age-related differences in the association between PA, OPA, and SCH

Subgroup analyses revealed a significant interaction between age and the associations of PA and OPA with SCH prevalence, with these associations being significant only in individuals aged <60 years. This age-related variation may be attributable to several physiological and metabolic factors. PA and OPA have been shown to reduce systemic inflammation, enhance insulin sensitivity, and modulate neuroendocrine pathways (20–25), all of which play crucial roles in thyroid homeostasis. However, in older adults, factors such as age-related metabolic decline, increased oxidative stress (26–28), and alterations in thyroid hormone metabolism may attenuate the protective effects of PA and OPA on SCH risk.

Additionally, the higher prevalence of comorbidities in older adults may introduce a more complex interplay between PA, OPA, and thyroid function, potentially diminishing the observed associations. These findings highlight the importance of considering age-specific strategies when evaluating the impact of PA and OPA on thyroid health.

### Potential mechanisms linking PA to thyroid function

The PA may influence thyroid function through several biological mechanisms. First, excess body weight and obesity are known risk factors for thyroid dysfunction, and PA plays a crucial role in regulating body weight and metabolism, thereby reducing the physiological burden on the thyroid gland (7–9). Additionally, adipose tissue acts as an endocrine organ, secreting hormones such as leptin and adiponectin, which are known to influence thyroid function (29, 30). Previous research has shown that obesity is associated with increased serum leptin levels, which may disrupt hypothalamic secretion of thyrotropin-releasing hormone, leading to elevated TSH levels (31, 32). Regular PA may help modulate these endocrine signals, thereby contributing to thyroid health maintenance.

Second, PA may protect thyroid health by reducing inflammation and enhancing immune function. Chronic inflammation is a well-established contributor to thyroid diseases, particularly autoimmune thyroiditis, a leading cause of SCH. Elevated levels of inflammatory markers such as C-reactive protein and fibrinogen have been shown to exacerbate thyroid dysfunction (16, 33). PA has been demonstrated to reduce these inflammatory markers and is associated with lower white blood cell, neutrophil, and lymphocyte counts, all of which play key roles in the immune response (16, 33).

Finally, PA may enhance thyroid function by improving circulation and increasing oxygen supply. Enhanced blood flow and oxygenation of thyroid tissues promote thyroid hormone synthesis and secretion (34, 35). Improved microcirculation not only facilitates nutrient delivery but also aids in waste removal, further supporting thyroid health. However, the precise mechanisms remain to be fully elucidated, and further research is needed to investigate the effects of PA intensity and duration on thyroid blood flow and metabolism.

### Clinical and public health implications

This study suggests that increased PA is associated with a lower prevalence of SCH, underscoring the potential role of PA in thyroid health. From a clinical and public health perspective, targeted screening for SCH may be particularly beneficial for individuals with lower PA levels, allowing for early detection and intervention. Furthermore, for those at higher risk of SCH, engaging in high-frequency and high-intensity OPA could serve as a potential preventive strategy. Healthcare professionals should integrate PA assessment into routine thyroid evaluations and encourage increased PA as part of preventive care strategies to support thyroid function and overall metabolic health.

### Limitations

Although this study provides meaningful findings, certain limitations must be considered. First, the cross-sectional design precludes the establishment of causal relationships between PA and SCH. While our findings indicate a significant association between higher PA levels and lower SCH prevalence, we cannot definitively conclude that PA directly reduces SCH risk. Additionally, the observed association may be influenced by reverse causality—for instance, individuals with SCH-related symptoms, such as fatigue, weight gain, or decreased exercise tolerance, may be less likely to engage in PA, rather than PA actively preventing SCH. This potential bidirectional relationship underscores the need for longitudinal studies to clarify the direction of causality.

Second, the wide age range of participants may introduce potential confounding effects. This constraint is inherent to the NHANES dataset and our inclusion criteria. Although we adjusted for age as a covariate in the regression models to mitigate its impact, residual confounding factors may still exist. Additionally, while a comprehensive set of potential confounders was included in our analysis, the influence of unmeasured or unaccounted factors cannot be entirely ruled out.

Third, the exclusion of participants with zero PA represents a potential limitation. Individuals with zero reported PA were found to have significantly higher age, elevated BMI, and a greater prevalence of metabolic comorbidities (e.g., hypertension, diabetes) (Supplementary Table S1). Their lack of PA may be more reflective of underlying health conditions rather than voluntary inactivity, making it difficult to disentangle the causal relationship between PA and SCH. While excluding this group reduces potential confounding, it may also limit the generalizability of our findings to populations with very low or no PA levels. Future studies should consider alternative analytical approaches to better account for this subset of individuals.

Lastly, the relatively small sample size of participants diagnosed with SCH may limit the generalizability and robustness of our findings. Given that SCH prevalence in the dataset was only 2.5%, the statistical power to detect certain subgroup differences may be constrained. Future research with larger sample sizes and longitudinal study designs is needed to confirm these associations, further explore causality, and better understand the underlying mechanisms linking PA to SCH.

## Conclusion

This study demonstrates that higher levels of PA, particularly at greater frequencies and intensities, are associated with a lower prevalence of SCH, especially in individuals aged <60 years. These findings suggest that both the duration and intensity of PA may contribute to a reduced SCH risk. However, given the cross-sectional design and the possibility of residual confounding, further longitudinal studies are needed to confirm these associations and explore the underlying mechanisms. Promoting regular, balanced PA may serve as a practical strategy for supporting thyroid health and SCH prevention.

## Data Availability

The datasets presented in this study can be found in online repositories. The names of the repository/repositories and accession number(s) can be found: www.cdc.gov/nchs/nhanes.

## Footnotes

^1^ www.cdc.gov/nchs/nhanes
