# Supplementary material for: Associations of physical activity type, intensity, and frequency with subclinical hypothyroidism: a cross-sectional analysis of NHANES 2007–2012
Source: Front Public Health. 2025 Jul 7;13:1499070. doi: 10.3389/fpubh.2025.1499070 (PMC12277262; doi:10.3389/fpubh.2025.1499070)
Supplement: Supplementary file 1 [file Table_1.docx]

Supplementary Material

# Supplementary Figures and Tables

Table S1. Baseline Characteristics of Participants Stratified by Physical Activity (PA = 0 vs. PA > 0)

|  | PA > 0 | PA = 0 | *P*-value |
| --- | --- | --- | --- |
| N | 6133 | 2244 |  |
| Age (years) | 44.99 ± 17.81 | 55.22 ± 18.11 | <0.001 |
| BMI (kg/m^2) (%) |  |  | <0.001 |
| < 25 | 2015 (33.18%) | 565 (25.92%) |  |
| 25-30 | 2087 (34.37%) | 695 (31.88%) |  |
| >= 30 | 1971 (32.46%) | 920 (42.20%) |  |
| Hypertension (%) |  |  | <0.001 |
| No | 4406 (71.84%) | 1223 (54.50%) |  |
| Yes | 1727 (28.16%) | 1021 (45.50%) |  |
| Diabetes (%) |  |  | <0.001 |
| No | 5566 (90.75%) | 1796 (80.04%) |  |
| Yes | 567 (9.25%) | 448 (19.96%) |  |

Continuous variables are presented as mean ± standard deviation (SD), while categorical variables are expressed as percentages (%). Abbreviation: PA, physical activity.
